# Supplementary material for: Control of protein activity by photoinduced spin polarized charge reorganization
Source: Proc Natl Acad Sci U S A. 2022 Aug 22;119(35):e2204735119. doi: 10.1073/pnas.2204735119 (PMC9436351; doi:10.1073/pnas.2204735119)
Supplement: Supplementary File [file pnas.2204735119.sapp.pdf]

Control of protein activity by photoinduced spin polarized charge reorganization

Shirsendu Ghosh[1,#,&], Koyel Banerjee-Ghosh[1,2,#], Dorit Levy[1], David Scheerer[1], Inbal Riven[1], Jieun Shin[3], Harry B. Gray[3], Ron Naaman[1,*] and Gilad Haran[1,*]

[1]Department of Chemical and Biological Physics, Weizmann Institute of Science, Rehovot, Israel

[2]Department of Chemistry, Indian Institute of Technology Hyderabad, Kandi, Sangareddy-502285, Telangana, India.

[3]Beckman Institute, California Institute of Technology, Pasadena, California 91125, United States

*Corresponding authors: Gilad Haran (email: gilad.haran@weizmann.ac.il) and Ron Naaman (email: ron.naaman@weizmann.ac.il).

[#]S.G. and K.B.-G. contributed equally to this work.

[&]S.G. current address: Department of Chemistry and Chemical Biology, Cornell University, Ithaca.

Supporting Information

Supporting Table 1: Analysis of antigen antibody association of Ru-tagged PGK with and without illumination

Mutant		NL	RCP	LCP	LP	RCP/NL	LCP/NL	LP/NL
**S290C/Ru/Surface**	Set1	37.5	38.7	41	41.7	1.03	1.09	1.11
	Set2	38.2	37.6	41.8	42.3	0.98	1.09	1.09
	Set3	36.6	36.9	40.7	41	1.01	1.11	1.12
**Q9C/Ru/Surface**	Set1	33.9	33.4	79.8	75.2	0.98	2.35	2.22
	Set2	29.2	34.7	83.8	72.7	1.19	2.87	2.49
	Set3	32.8	35.5	82.1	73.7	1.08	2.50	2.25
**Q9C/Ru/Glass**	Set1	26	--	--	66.6	--	--	2.56
	Set2	25.2	--	--	59.5	--	--	2.36
	Set3	27.3	--	--	64.2	--	--	2.35
**Q9C/No Ru/Glass**	Set1	25.2	--	--	27.3	--	--	1.08
	Set2	26	--	--	25.1	--	--	0.96
	Set3	27	--	--	25.2	--	--	0.93

1. All values are average number of molecules obtained from counting of at least 9 different regions of individual samples of each set.
2. NL- no light; RCP- right circularly polarized light; LCP- left circularly polarized light; LP- linearly polarized light.

Supporting Table 2: Analysis of activity assays of Ru-tagged PGK with and without illumination

**Mutant**		**NL**	**RCP**	**LCP**	**LP**	**NL/RCP**	**NL/LCP**	**NL/LP**
**S290C**	Set1	-0.017	-0.014	-0.008	-0.006	1.21	2.12	2.83
	Set2	-0.011	-0.009	-0.004	-0.003	1.22	2.75	3.66
	Set3	-0.010	-0.009	-0.004	-0.003	1.11	2.5	3.33
**Q9C**	Set1	-0.018	-0.018	-0.010	-0.009	1	1.8	2
	Set2	-0.018	-0.014	-0.010	-0.009	1.28	1.8	2
	Set3	-0.013	-0.013	-0.007	-0.0068	1	1.86	1.9

1. All slopes are given in units of absorbance change per minute. Note that slopes vary between different samples due to differences in the surface densities of the proteins; however, the ratios are similar within experimental error.
2. NL- no light; RCP- right circularly polarized light; LCP- left circularly polarized light; LP- linearly polarized light.

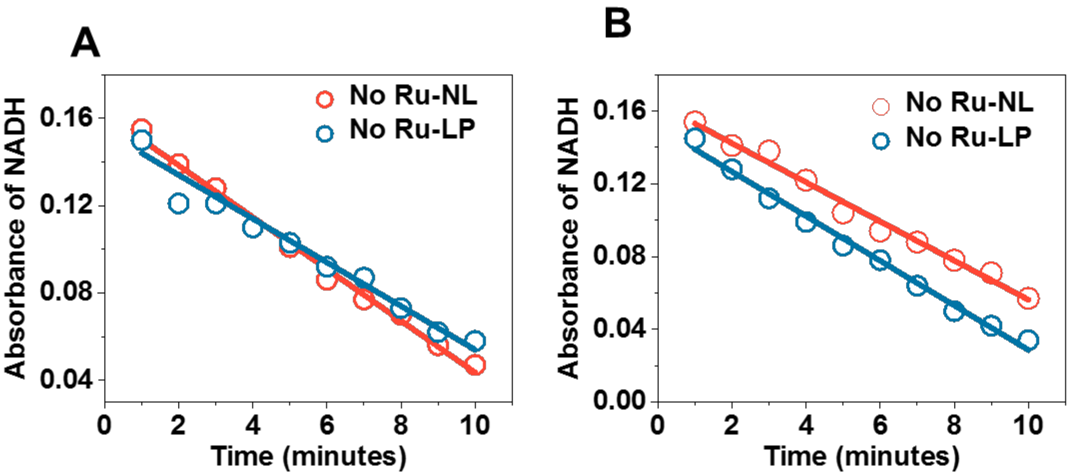

Supporting Figure 1. In the absence of Ru, no effect of light is observed on the enzymatic kinetics of PGK. A. S290C. **B.** Q9C. NL- no light. LP- linearly polarized light. Experiments were repeated twice.

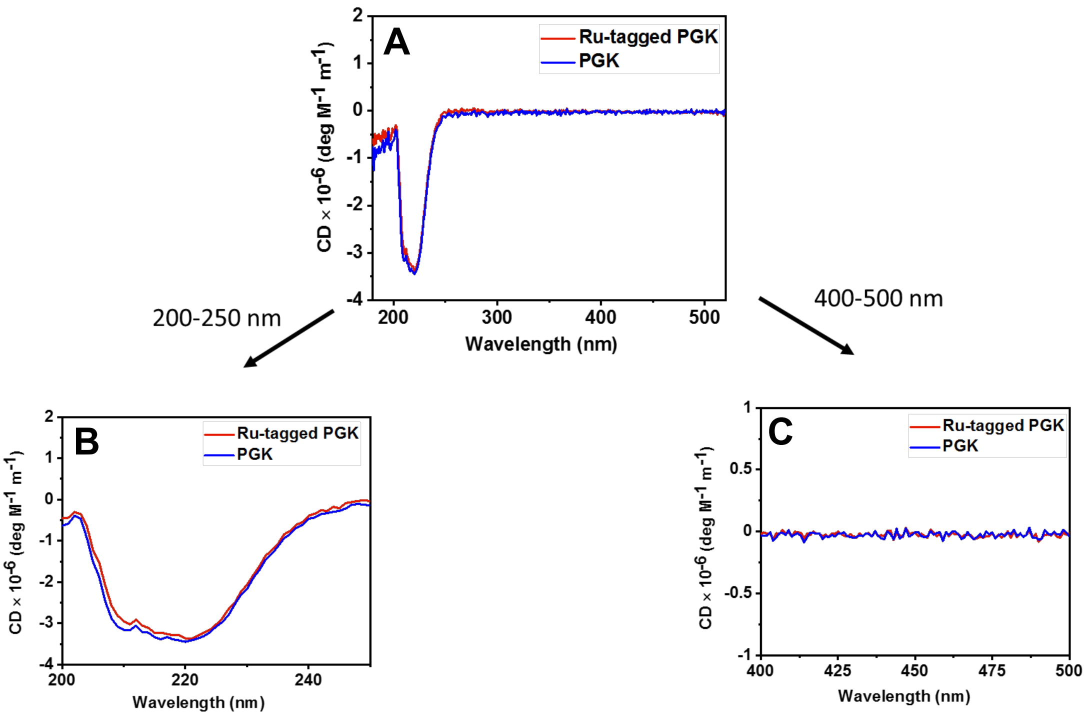

Supporting Figure 2. Labeled protein did not show any optical activity at the wavelength of absorption of the Ru group: A. Circular dichroism spectra of unlabeled and Ru labelled PGK. **B.** Zoomed section of the spectral region 200-250 nm of A. **C.** Zoomed section of the spectral region 400-500 nm of A. Experiments were repeated twice.
